# Effectiveness of Ryokeijutsukanto in the Treatment of Benign Paroxysmal Positional Vertigo (BPPV)

**DOI:** 10.7759/cureus.87161

**Published:** 2025-07-02

**Authors:** Fumiyuki Goto, Shoji Kaneda, Koichiro Wasano

**Affiliations:** 1 Otolaryngology - Head and Neck Surgery, Tokai University School of Medicine, Isehara, JPN; 2 Otolaryngology- Head and Neck Surgery, Tokai University, Isehara, JPN

**Keywords:** benign paroxysmal positional vertigo (bppv), brandt-daroff method, drug therapy, herbal drug, ling gui zhu gan tang

## Abstract

Background

Benign paroxysmal positional vertigo (BPPV) is the most common acute vertigo disorder caused by otolith displacement in the inner ear. Recurrence, particularly in older adults, poses a significant clinical challenge.

Objectives

This study evaluates the therapeutic effects of ryokeijutsukanto, a Kampo medicine, on BPPV when used adjunctively with the Brandt-Daroff (BD) method.

Methods

A prospective cohort study was conducted following the STROBE (Strengthening the Reporting of Observational Studies in Epidemiology) guidelines. Forty-eight BPPV patients were divided into three groups: Group A (BD method only, N=8), Group B (BD method + Kampo, N=11), and Group C (BD method + betahistine 36 mg, N=29). As Kampo, ryokeijutsukanto 7.5 mg was given three times a day before meals, divided into three doses. The BD method was performed daily, and the outcomes were measured by subjective improvement and positional nystagmus resolution.

Results

Positional nystagmus resolution occurred in 38.3 ± 34.5 days (Group A), 30 ± 30.2 days (Group B), and 20.8 ± 21.4 days (Group C), with no statistically significant differences between groups. Subjective improvements were observed in 55% (6/11) of patients in Group B.

Conclusions

While ryokeijutsukanto did not significantly impact nystagmus resolution times, it enhanced subjective outcomes and adherence, suggesting its potential as an adjunctive therapy in BPPV management.

## Introduction

Benign paroxysmal positional vertigo (BPPV) is the most common cause of acute vertigo and is typically managed with canalith repositioning techniques such as the Brandt-Daroff (BD) method [1]. Although effective in resolving the underlying otolithic dislocation, some patients continue to experience residual dizziness, emotional distress, and anxiety that can persist for weeks following successful repositioning maneuvers [2]. These limitations underscore the need for adjunctive therapeutic strategies to improve recovery and adherence.

In recent years, Kampo medicine, traditional Japanese herbal medicine, has attracted attention as a potential integrative treatment for dizziness-related disorders. Kampo formulations such as hangebyakujutsutemmato have demonstrated potential efficacy in managing persistent postural-perceptual dizziness (PPPD) [3,4], while Yokukansan has been shown to reduce chronic dizziness accompanied by emotional instability [5]. These findings support the use of Kampo formulas targeting both somatic and psychological dimensions of dizziness.

Ryokeijutsukanto is a Kampo formula traditionally prescribed for symptoms such as dizziness, fatigue, edema, and anxiety. Its constituent herbs include Poria Sclerotium (4.0 g), Cinnamomi Cortex (3.0 g), Atractylodis Lanceae Rhizoma (4.0 g), and Glycyrrhizae Radix (2.0 g). Pharmacological studies have suggested that these herbs exert sedative, anxiolytic, and diuretic effects, which may be beneficial for managing both physiological and psychosomatic aspects of vertigo. For example, Cinnamomi Cortex has been associated with GABAergic modulation and reduced stress responses, while Poria is known for anti-inflammatory and anxiolytic properties.

Given the multifactorial nature of vertigo and the interplay between vestibular dysfunction and psychological stress, we hypothesized that ryokeijutsukanto might enhance the therapeutic outcomes of BPPV treatment by improving subjective symptoms and promoting adherence to physical maneuvers. Despite its long-standing use, evidence supporting the role of ryokeijutsukanto in BPPV management is lacking. Therefore, the present study aims to evaluate the adjunctive use of ryokeijutsukanto in patients undergoing BD therapy for BPPV, with a focus on subjective improvement, adherence, and resolution of positional nystagmus.

## Materials and methods

This prospective cohort study was conducted at Tokai University Hospital in accordance with the STROBE (Strengthening the Reporting of Observational Studies in Epidemiology) guidelines [6]. Ethical approval was obtained from the Tokai University Hospital Institutional Review Board (Approval No. 24R086-001), and written informed consent was obtained from all participants prior to enrollment. The study adhered to the principles outlined in the Declaration of Helsinki.

A total of 48 patients (17 men and 31 women; mean age: 65.6 ± 10.6 years) diagnosed with posterior semicircular canal benign paroxysmal positional vertigo (BPPV) were recruited. Participants were non-randomly assigned to three treatment groups based on physician recommendation and patient preference. Group A (n = 8) received the Brandt-Daroff (BD) method only, Group B (n = 11) received the BD method in combination with ryokeijutsukanto, and Group C (n = 29) received the BD method with betahistine 36 mg per day. Ryokeijutsukanto extract granules (7.5 g/day; Tsumura & Co., Tokyo, Japan) were administered orally in three divided doses before meals. A daily dose (7.5 g) of ryokeijutsukanto (TJ-26, Tsumura & Co., Tokyo, Japan) extract granules is equivalent to the following crude herbs: Poria Sclerotium 4.0 g, Cinnamomi Cortex 3.0 g, Atractylodis Lanceae Rhizoma 4.0 g, and Glycyrrhizae Radix 2.0 g, known for their potential diuretic and anxiolytic effects.

The BD method was performed daily by all participants as instructed. The primary outcomes included time to resolution of positional nystagmus, measured in days, and subjective improvement in vertigo symptoms. Subjective improvement was categorized as “effective,” “not effective,” or “medication intolerance due to side effects.”

Statistical analysis

All statistical analyses were conducted using IBM SPSS Statistics version 28.0 (IBM Corp., Armonk, NY, USA). Continuous variables (e.g., duration to resolution of positional nystagmus) were summarized as means ± standard deviations. Categorical variables (e.g., subjective improvement) were reported as frequencies and percentages. Between-group comparisons of continuous outcomes were performed using one-way analysis of variance (ANOVA). Where the ANOVA indicated overall significance, Bonferroni-corrected pairwise comparisons were planned. Categorical outcomes were compared using the chi-square test or Fisher’s exact test, as appropriate. A p-value <0.05 was considered statistically significant. Due to the exploratory design and relatively small sample size, adjustments for potential confounding variables (age, sex, baseline severity, and anxiety) were not statistically controlled, though these factors were considered in the interpretation of findings.

Given the exploratory design and modest sample size (Group A: N=8, Group B: N=11, Group C: N=29), a post hoc power analysis was performed for the primary continuous outcome (time to resolution of positional nystagmus). Based on the observed standard deviation (~30 days) and an expected minimum clinically relevant difference of 20 days between groups (effect size d ≈0.67), a conventional two-sided ANOVA with alpha=0.05 achieved an estimated power of approximately 0.43. Therefore, the study was underpowered to detect small to moderate intergroup differences. Future randomized studies with larger samples (at least n=36 per group) would be required to achieve 80% power to detect similar effect sizes, assuming comparable variance estimates, consistent with sample size calculations reported in prior BPPV intervention studies [7].

## Results

The demographic and baseline characteristics of the 48 enrolled patients are summarized in Table 1.

**Table 1:** Baseline Characteristics of the Study Participants Note: Comorbidities and disease severity data were not collected in this study. BD: Brandt-Daroff method

Characteristics	Group A (BD only) N=8	Group B (BD + Kampo) N=11	Group C (BD + Betahistine) N=29	Total N=48
Age, mean ± SD (years)	67.2 ± 9.8	66.5 ± 11.2	64.8 ± 10.4	65.6 ± 10.6
Sex, n (%)				
- Male	3 (37.5%)	4 (36.4%)	10 (34.5%)	17 (35.4%)
- Female	5 (62.5%)	7 (63.6%)	19 (65.5%)	31 (64.6%)

**Figure 1:**
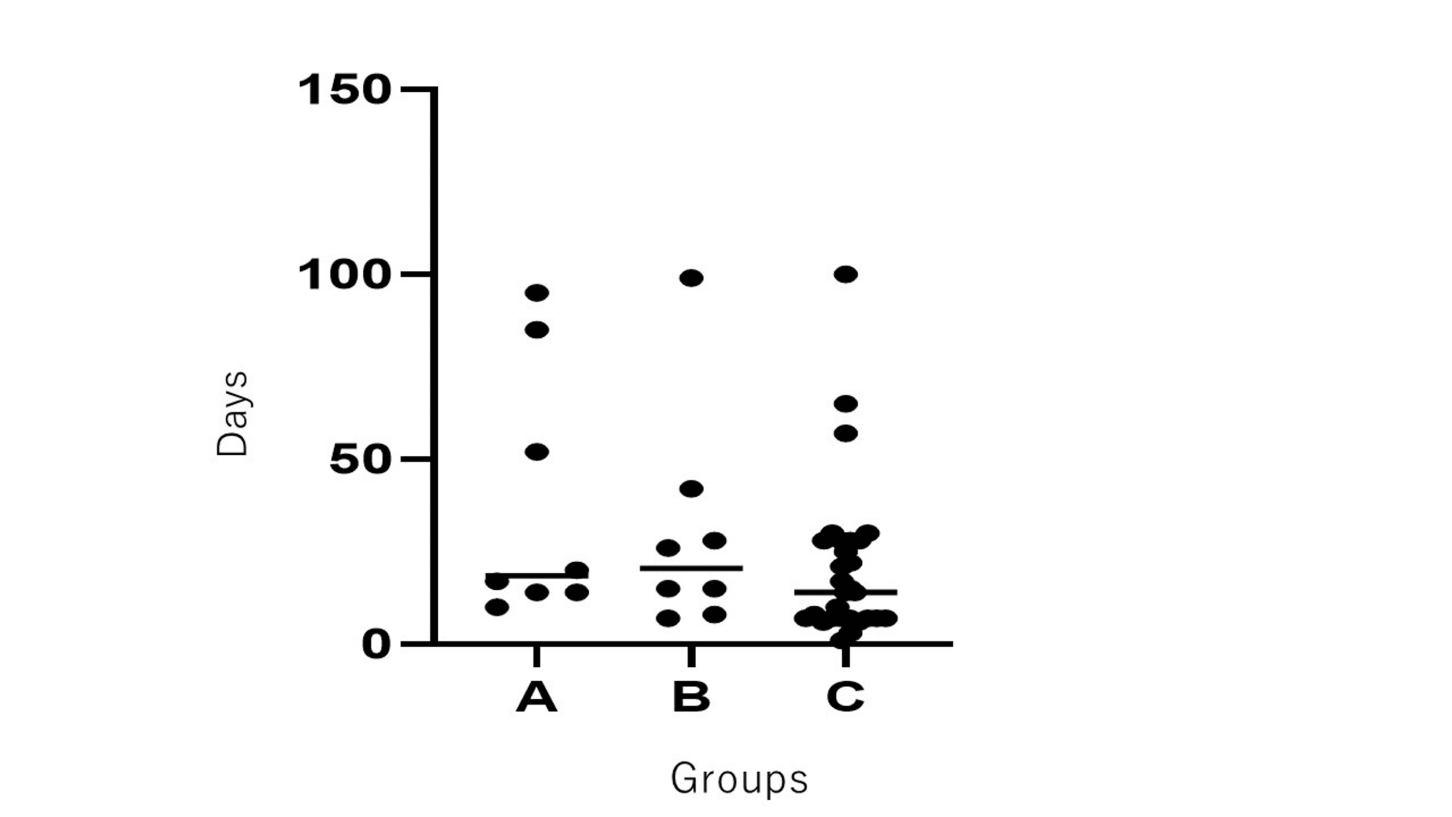
Duration of Positional Nystagmus Resolution Across Groups We compared the duration of positional nystagmus among the three groups: Group A (BD method only, Nt=8), Group B (BD method + Kampo, N=11), and Group C (BD method + betahistine 36 mg, N=29). The vertical axis represents the number of days until the resolution of positional nystagmus. Each bar indicates the median duration for each group. BD: Brandt-Daroff method

The mean duration to resolution of positional nystagmus was 38.3 ± 34.5 days in Group A (N=8), 30.0 ± 30.2 days in Group B (N=11), and 20.8 ± 21.4 days in Group C (N=29) (Figure 1).

Although Group C demonstrated the shortest recovery period, ANOVA revealed no statistically significant differences among the three groups (F = 1.05, p = 0.36).

**Figure 2:**
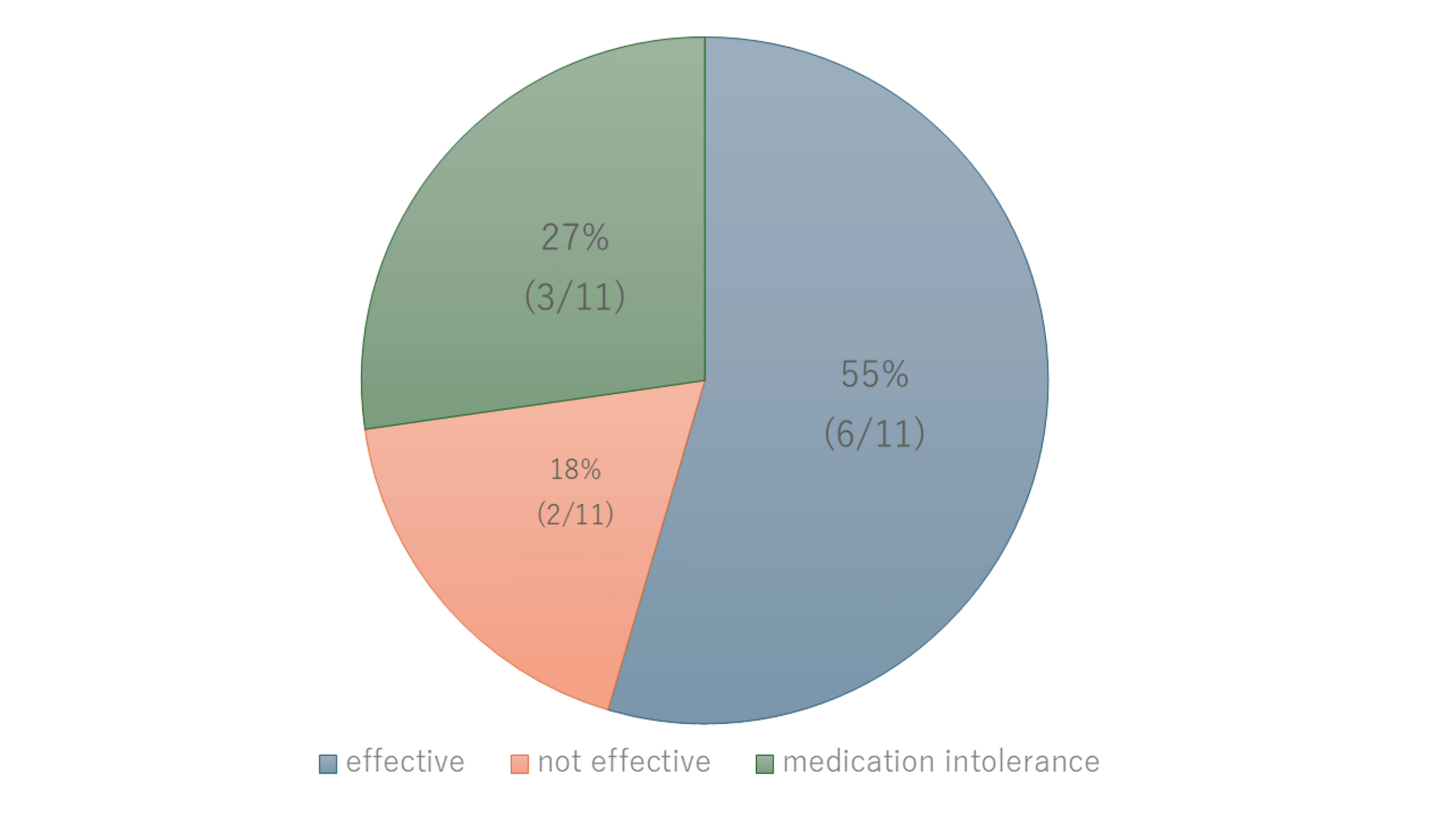
Subjective Effect in Group B (N=11) Subjective efficacy was classified into three groups: effective, not effective, and medication intolerance due to side effects.

Within Group B, three patients were unable to continue the medication. Subjective improvement was reported by six out of 11 patients in Group B (55%) (Figure 2), compared to four out of eight patients in Group A (50.0%) and 21 out of 29 in Group C (72.4%).

A chi-square test comparing the proportions showed no significant differences (χ² = 1.14, df = 2, p = 0.56, Cramér’s V = 0.15). Within Group B, all eight patients who completed the treatment expressed a desire to continue the medication at the follow-up visit two weeks later. Even after the resolution of nystagmus, three patients wished to continue the medication.

No participants reported adverse effects leading to discontinuation in Groups A or C. In Group B, one patient (9.1%) experienced mild gastrointestinal discomfort but continued therapy. Adherence to the BD method was notably higher in Group B, particularly in those reporting symptom relief, suggesting a potential motivational benefit from symptom reduction.

While the use of ryokeijutsukanto did not significantly accelerate the resolution of nystagmus, its positive impact on subjective outcomes and treatment adherence underscores its potential value as an adjunctive therapy in BPPV management.

## Discussion

This study provides insights into the potential benefits of ryokeijutsukanto as an adjunctive therapy for BPPV. Despite the lack of statistically significant differences in nystagmus resolution times, the Kampo medicine demonstrated notable improvements in subjective outcomes. The observed trend suggests potential clinical benefits, which warrant further investigation. Such enhancements may stem from its anxiolytic effects, particularly beneficial for managing the psychological distress often associated with vertigo [8]. The inclusion of cinnamon twig and poria, known for their sedative and diuretic properties, respectively, aligns with findings from studies on holistic approaches to vestibular dysfunction [9,10].

Moreover, adherence to the BD method was higher in patients using ryokeijutsukanto, likely due to reduced anxiety and improved overall well-being. Enhanced adherence is a critical factor in achieving therapeutic success, especially in conditions requiring consistent physical maneuvers [11]. This finding is consistent with previous research emphasizing the role of patient-centered care in vertigo management [12].

However, this study was not randomized and was conducted at a single institution with a relatively small sample size. These factors may have introduced confounding variables such as age, sex, and baseline severity of BPPV, potentially limiting the generalizability of the findings. Additionally, outcome assessment was based on subjective reports, which may introduce reporting bias. Another important limitation is the short follow-up period. While symptom resolution and treatment adherence were monitored over a brief timespan, the long-term effects of ryokeijutsukanto on recurrence rates, functional recovery, and psychological well-being remain unknown.

To address these limitations, future investigations should employ well-designed randomized controlled trials (RCTs) with larger sample sizes, stratified randomization, longer observation windows, and robust blinding procedures to provide a more rigorous evaluation of the therapeutic effects of ryokeijutsukanto. Exploring the biochemical mechanisms underlying its efficacy could also yield deeper insights. For example, its influence on neurotransmitter pathways, particularly those regulating anxiety and balance, warrants further exploration [13].

Another area of interest is the potential application of Kampo medicine in other vertigo-related conditions, such as Meniere’s disease. Moreover, adjunctive betahistine treatment has shown promise in reducing symptom duration in BPPV, especially when combined with physical maneuvers [14,15]. The overlap in pathophysiological mechanisms suggests that similar therapeutic benefits could be achieved. Additionally, assessing the long-term outcomes of ryokeijutsukanto use, including its role in preventing recurrence, could enhance its clinical applicability.

Despite these limitations, our study underscores the potential of integrating Kampo medicine into conventional BPPV treatment paradigms. Ryokeijutsukanto, with its historical use and pharmacological plausibility, represents a promising adjunct for patients who experience residual dizziness or struggle with adherence to physical therapies. Importantly, the observed trends in symptom improvement and adherence support the hypothesis that addressing psychological distress may enhance overall treatment outcomes in vertigo [15].

## Conclusions

In conclusion, while our findings do not establish definitive efficacy, they provide a basis for hypothesis generation and future investigation. This study underscores the potential of ryokeijutsukanto as a supplementary therapy for BPPV, with observed benefits in improving subjective symptoms and treatment adherence. The incorporation of culturally relevant, low-risk adjunctive therapies aligns with the principles of patient-centered care and integrative medicine. Continued research, including well-designed randomized controlled trials, is warranted to substantiate these preliminary insights, refine our understanding of their mechanisms, and develop comprehensive treatment strategies that may support broader clinical adoption.
